# Editorial: Model organisms in renal pharmacology: 2022

**DOI:** 10.3389/fphar.2023.1139806

**Published:** 2023-02-07

**Authors:** Ayman M. Mahmoud, Weiqiang Lin, Vikram Patial, Rajesh Nachiappa Ganesh, Pragasam Viswanathan

**Affiliations:** ^1^ Department of Life Sciences, Faculty of Science and Engineering, Manchester Metropolitan University, Manchester, United Kingdom; ^2^ Physiology Division, Zoology Department, Faculty of Science, Beni-Suef University, Beni-Suef, Egypt; ^3^ International Institutes of Medicine, The Fourth Affiliated Hospital, Zhejiang University School of Medicine, Jinhua, Zhejiang, China; ^4^ Pharmacology and Toxicology Laboratory, Dietetics & Nutrition Technology, CSIR-Institute of Himalayan Bioresource Technology, Palampur, India; ^5^ Department of Pathology, JIPMER, Puducherry, India; ^6^ Department of Pathology and Genomic Medicine, Houston Methodist Hospital and Academic Institute, Houston, TX, United States; ^7^ Renal Research Lab, Centre for Bio-Medical Research, School of Bio-Sciences and Technology, Vellore Institute of Technology, Vellore, Tamil Nadu, India

**Keywords:** chronic kidney disesase, nephropathy, renal fibrosis, animals models, extracellular vescicles

Chronic kidney disease (CKD) is a leading public health problem that affects a significant proportion of the population worldwide. It is a complex multifactorial disease and approximately 13% of the world population are affected by this disease (Evans et al., 2022). The number of patients with CKD reached 843.6 million in 2017 and is subject to increase over the upcoming years (Jager et al., 2019). Although the prevalence of CKD is high, only 10% of the population at high risk of CKD are aware of their disease status (Ene-Iordache et al., 2016). Hypertension, obesity, cardiovascular disease (CVD) and type 2 diabetes are among the factors contributing towards CKD (Evans et al., 2022). The progression of CKD is associated with socioeconomic burden and serious clinical outcomes, including end-stage renal disease, renal failure and CVD (Evans et al., 2022). CKD is associated with increased cardiovascular morbidity and mortality (Jager et al., 2019) and its early detection and the development of effective preventative and therapeutic strategies to attenuate its progression are of utmost importance.

The use of experimental models represents a valuable tool to understand the underlying pathophysiological mechanisms of kidney diseases and exploring novel therapeutic targets. Model organisms, in particular rats and mice, represent an invaluable resource for both fundamental and applied research, allowing prediction studies, modeling, and the identification of action mechanisms. Drug development requires tests on model organisms before the clinical trials and this makes these organisms essential for progress within the field.

This Research Topic encompasses two research articles and two review articles. These articles introduced cutting-edge research on diabetic nephropathy (DN) and the role of cellular senescence in CKD. In addition, the role of extracellular vesicles (EVs) in acute kidney injury (AKI), CKD, DN and renal tumors has been discussed. However, drug/chemical-induced nephrotoxicity and other models of AKI were not discussed in these articles. The guest editors are pleased to present a compendium of these articles as follows:

In the research article by Hou et al., the investigators utilized the connectivity map (CMap) with renal tubulointerstitial transcriptomic profiles of biopsy-proven early- and late-stage DN, a leading cause of CKD, to identify novel treatment candidates. The therapeutic potential of the suggested candidate drugs was evaluated *in vivo* in streptozotocin-induced CD-1 mice, and *in vitro* utilizing HK-2 cells and immortalized bone marrow-derived macrophages (iBMDMs). CAY10603, a specific inhibitor of histone deacetylase 6 (HDAC6), was defined as a potential drug and the experimental findings showed its ameliorative effect on renal dysfunction, inflammatory cell infiltration, tubular injury and tubulointerstitial fibrosis in diabetic mice. These effects were associated with the inhibition of NLRP3 and pyroptosis as revealed by the studies on HK-2 cells and iBMDMs.

In another article, Wang et al. summarized and discussed the findings of the studies showing the effects of the active constituents of *Rheum ribes* on key signaling pathways implicated in the pathogenesis of renal fibrosis. The conducted analysis revealed the beneficial effects of *Rheum ribes* constituents against the development of renal fibrosis and their value as drug candidates.

Etiology, pathological changes, phenotype, and outcome are among the similarities that renal senescence shares with CKD. Given these numerous similarities, is not clear whether renal senescence is a trigger or a consequence of CKD. In this review article, Zhao et al. summarized the common features and the interaction between CKD and renal senescence, and the therapeutic approaches, including senolytics, senomorphics, and immunomodulation. This review article highlighted the value of renal senescence as a promising target for therapeutic intervention of CKD.

Xiang et al. summarized the latest studies showing the role of EVs in renal diseases and highlighted their value as therapeutic targets. EVs are cell-derived membrane structures released by most cells and play a role in cell-cell communication and as vehicles for information transmission. The association between EVs and renal diseases has been acknowledged. This review highlighted the updated progress in understanding the role of EVs in the progression of renal diseases and the possible therapeutic application of stem cell-derived EVs.

The editors anticipate the published articles in this Research Topic to be of interest to the readers and expect researchers to benefit from achieving further progress in understanding the mechanisms underlying renal diseases and the development of novel therapeutics.
